# Little All Children in Focus (Little ACF) – evaluation of a parenting support programme for parents of children aged 1–2 years in Sweden: a randomised controlled trial

**DOI:** 10.1186/s12889-026-27733-2

**Published:** 2026-05-19

**Authors:** Anna Edenius, Lene Lindberg, Johan Åhlén, Pia Enebrink, Malin Bergström

**Affiliations:** 1 Department of Global Public Health, Karolinska Institutet, Stockholm, 171 77 Sweden; 2 Center for Epidemiology and Community Medicine, Stockholm County, Sweden; 3 Department of Clinical Neuroscience, Karolinska Institutet, Stockholm, 171 77 Sweden

**Keywords:** Emotion regulation, Mental health, Promotion, Universal parenting support, Group intervention, Toddler

## Abstract

**Introduction:**

The first two years of life affect health over the life course. Universal parenting support programmes may promote child health during these years but there is a lack of such evidence-based programmes. Our aim was to study the direct effects of a structured 4 sessions universal parenting programme for parents with children 1–2 years old, Little All Children in Focus, in Sweden.

**Methods:**

A two-arm parallel randomised controlled superiority trial with 832 Swedish-speaking parents was conducted to evaluate the effectiveness of the Little All Children In Focus programme in comparison with an active control receiving four digital lectures on child development and parenthood. The participants completed questionnaires at baseline and post intervention measuring the primary outcomes: parenting self-efficacy and stress, coparenting quality, emotion regulation for parents and emotion regulation strategies for their children, and secondary outcome: child socioemotional development. A third research question concerned whether the intervention could engage and retain parents in groups. An intention-to-treat approach was applied using linear mixed models for analysing the effectiveness of the programme.

**Results:**

Parents who participated in the programme demonstrated greater improvements in supportive emotion regulation strategies compared to the active control ($\beta$ = 3.0, CI = 0.9 – 5.0), while no notable group differences emerged across the remaining outcome measures. The program demonstrated high levels of parental engagement and retention in group settings.

**Conclusions:**

Little All Children in Focus demonstrates potential in promoting supportive parenting emotion regulation strategies among Swedish speaking parents to 1–2-year-olds. The intervention also seems to have potential to engage parents. However, further evaluations must be conducted to provide a deeper understanding of the long-term impact of this universal parenting support programme.

**Trial registration:**

The study protocol https://rdcu.be/eAxn2 was published at www.clinicaltrials.gov with registration number: NCT05445141 (registered on July 06, 2022).

**Supplementary Information:**

The online version contains supplementary material available at 10.1186/s12889-026-27733-2.

## Introduction

During the first years of life, all aspects of child development progress rapidly, influencing mental and physical health, as well as overall well-being throughout life [1]. The quality of the early parent–child relationship is crucial for the child’s well-being [2]. Several parenting factors, such as positive perceptions toward the child’s intentions, low parenting stress, and a supportive coparenting relationship, as well as contextual resources such as strong social support, have been shown to facilitate responsive parenting and to be protective against child behavioural problems [3]. Furthermore, a strong sense of parenting self-efficacy enhances a parents’ confidence in managing everyday challenges during toddlerhood [4, 5]. A central aspect of parenting during the first three years is to support the development of the child’s emotion regulation through practices such as affective mirroring, verbalising emotions, modelling calm responses, and showing positive emotions towards the child [6–8]. Parents’ own emotion regulation capacities and their strategies for responding to children’s negative emotions are therefore critical, as they shape the child’s opportunities to learn adaptive regulation [9].

During toddlerhood, children’s increased mobility, autonomy, and willpower require new parenting skills. Parental support to facilitate the development of such skills may therefore be beneficial [2]. Universal parenting support programmes are directed towards the whole population, without identified risk factors or problems, and are typically associated with smaller effect sizes compared to targeted programmes [10]. These interventions are more frequently developed for parents of children 3 years and up, whereas comparatively fewer target parents of the youngest children aged 1–2 years [11–13].

A systematic review and meta-analysis of parenting interventions to promote child development during the first three years of life found positive effects on parenting practices and parent–child relationships. Benefits were also observed for children’s mental health, including socioemotional development and reductions in behavioural problems [14]. Two Cochrane systematic reviews examined group-based parent training programmes targeting parents of children primarily in the age range 0–6 years. The results indicated short-term improvements in children’s emotional and behavioural adjustment, as well as in parental psychosocial health, including stress, self-confidence, and the coparenting relationship [15, 16]. The quality of the coparenting relationship has been highlighted as a protective factor for both parental well-being and child adjustment, even during the first years of life [17, 18].

Evidence indicates that parenting programmes for parents of children in early childhood (0–7 years) achieve greater impact on both child and parenting outcomes when they include components that promote responsive caregiving, emotional communication, parenting consistency, and offer opportunities to practice parenting skills during sessions [10, 14, 19]. Of note, these previously mentioned meta-analyses and systematic reviews combine results across different populations (e.g., universal samples, children with elevated symptoms, and groups at heightened risk) [10, 14–16, 19]. As an example of this broader evidence base, there is accumulating evidence for nurse home visiting programmes during the first two years of life; however, these interventions are typically selective and extensive, targeting families with multiple risk factors [20, 21]. Research on universal parenting programmes for young children 0–3 years remains scarce, although a few programmes have been evaluated and shown promising results on both parenting behaviours and competencies, as well as children’s behaviours and functioning [11, 22, 23]. Taken together, existing studies provide encouraging indications, but the overall knowledge base remains limited, particularly regarding universal parenting programmes for parents of toddlers aged 1–2 years. This highlights the need to examine outcomes that capture both parental aspects and child socioemotional development. Whether parenting programmes are able to retain and engage parents in groups is another key indicator of feasibility and long-term impact, but this aspect remains underrepresented in research on universal interventions [13, 24].

All Children in Focus (ACF) is a structured universal parenting support programme developed and evaluated in Sweden for parents with children between 3–12 years of age. A randomised controlled trial (RCT) found that ACF increased parents’ self-efficacy and the well-being of the child [12]. A recent follow-up study confirmed that parents receiving ACF reported higher levels of emotion regulation skills and positive strategies than parents in the control condition [25]. The initial evaluation of the ACF programme did not include children aged 1–2 years, and a new version of the programme has been developed to specifically address the needs of this age group: Little All Children in Focus (Little ACF) [26].

Embedding interventions within existing services that provide support to parents may reduce stigma and allow participation of parents who would not be reached by targeted interventions [27, 28]. In Sweden, the Child Health Services (CHS) is well-developed, free of charge, and reaches about 97% of all children from birth up to five years of age [29]. During the child’s first year, contact with CHS is frequent, with most visits scheduled before 10 months of age. After this period, the support gradually becomes sparse, with routine appointments at 12 and 18 months and then not again until the child turns 3 years [30]. This results in a gap in support for families during this stage. Therefore, the CHS was considered an ideal platform for recruiting and providing families with structured universal parenting support at this early stage.

### Aim and research questions

The aim of this trial was to study effects of a universal group-based intervention for parents of children aged 1–2 years, the Little ACF programme. Our specific research questions were whether Little ACF could:

- *Primary outcomes* (parental measures): affect parenting self-efficacy, parenting stress, coparenting quality, parents’ emotion regulation, and parents’ strategies for regulating their children’s negative emotions,
- *Secondary outcome* (child measure): affect children’s social and emotional development,
- *Feasibility outcome:* engage and retain parents in groups.

## Methods

### Study design

We conducted a two-arm parallel randomised controlled superiority trial, to evaluate the effectiveness of Little ACF compared with an active control group receiving four digital lectures on child development [26]. The allocation ratio applied was 1:1. Data were collected at baseline and post-intervention through digital questionnaires completed by participating parents. The trial was designed and reported in accordance with the CONSORT guidelines.

### Sample size and power calculation

The previous RCT of the ACF programme for parents of children aged 3–12 found an effect size (Cohen's *d*) of 0.30 in improving parenting self-efficacy compared to a waitlist [12]. In the current study, we anticipated a slightly smaller effect size (*d* = 0.25) since we compared the Little ACF with digital lectures. Using an alpha level of 0.05 and a power of 80%, a total of 251 children per group were required. Although multiple parents per child were eligible to participate, the sample size analysis was based on a conservative approach of only one parent per child. Considering the expected dropout rate of 25%, it was necessary to enrol parents of 670 children. While the study was ongoing the researchers found a higher attrition than expected. As a result, the trial was extended by another year, and more families were included than originally planned.

### Participants

#### Group leaders

We recruited 47 group leaders, working in pairs with one group leader from the CHS and one from the municipality or civil society. Group leaders from CHS were pediatric nurses, district nurses, or held both qualifications, with a minimum of five years’ experience working in child health clinics. Group leaders from municipal or civil society settings were parenting advisors or early childhood educators, all of whom worked professionally with young children. With 5–8 parents per Little ACF group, approximately 60–70 groups were needed, and each group leader was expected to facilitate at least three groups. The management at each CHS unit had to approve participation so the unit had sufficient capacity to recruit at least 30 families during the study period. Most participating CHS units were located in the Region of Stockholm, the rest in other urban and rural areas in Sweden. An invitation letter for participation in the study was sent to all CHSs in the region of Stockholm in April 2022 and resulted in the inclusion of 28 group leaders. A year later an additional 19 group leaders from other regions were recruited.

#### Parents

All Swedish speaking parents with 1–2-year-old children who were registered at a participating CHS were eligible for the study. The CHS nurses’ assessments of parents’ proficiency in Swedish was based on their previous frequent contacts with the families. All the child’s caregivers were encouraged to participate.

**Fig. 1:**
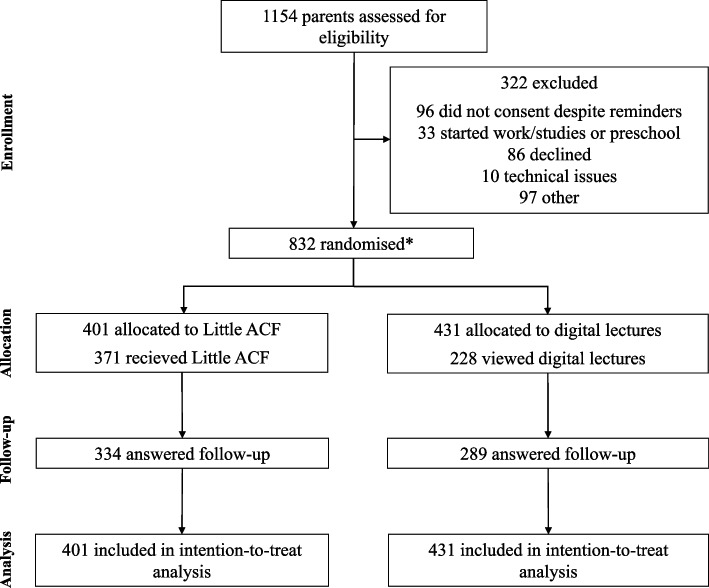
Flowchart of parents’ flow during the trial. Note: *While the flowchart refers to parents for clarity and brevity, it is the children who are randomised in this trial. Further details are provided in the Methods section

A total of 832 parents of 769 children completed the baseline questionnaire, and of these 642 (77%) returned the post-intervention questionnaire. A total of 322 eligible parents were excluded, with the most frequent reasons being that parents changed their minds about participation or did not have enough time (Supplementary Table S1, Additional File 1). Fig. 1 presents a flowchart illustrating the participant timeline and attrition in the study.

We found no differences in baseline characteristics between participants in the Little ACF and the control group (Table 1). The average parental age was 34 years for mothers and 37 years for fathers, 70% of the parents had a university education, and the majority, about 80% of the parents, were born in Sweden. Approximately 70% of participants were mothers and 30% were fathers. Among the children, the mean age in both groups was almost 16 months, and the majority (nearly 95%) lived with both parents.

**Table 1:** Sociodemographic characteristics of parents and children, shown in frequencies, percentages, means, and standard deviations (*N* = 832)

	All (*N* = 832)	Intervention (*n* = 401)	Control (*n* = 431)
	% (N)	% (n)	% (n)
Respondent			
Mother	69.5 (578)	68.6 (275)	70.3 (303)
Father	30.5 (254)	31.4 (126)	29.7 (128)
Parental age in years (mean, SD)			
All	35.5 (5.0)	35.6 (4.9)	35.4 (5.1)
Mothers	34.8 (4.8)	35.0 (4.7)	34.7 (5.0)
Fathers	37.1 (5.0)	37.2 (5.0)	37.1 (5.1)
Parental country of birth			
Sweden	81.6 (679)	83.5 (335)	79.8 (344)
Other country	18.4 (153)	16.5 (66)	20.2 (87)
Parental educational level			
Elementary school or lower	2.6 (22)	2.5 (10)	2.8 (12)
High school	27.4 (228)	27.9 (112)	26.9 (116)
University/college	70.0 (582)	69.6 (279)	70.3 (303)
Previous parental group			
Yes	35.9 (299)	36.2 (145)	35.7 (154)
Child’s gender			
Girl	52.0 (433)	54.1 (217)	50.1 (216)
Boy	48.0 (399)	45.9 (184)	49.9 (215)
Child’s age in months (mean, SD)	15.8 (5.7)	15.6 (5.6)	15.9 (5.9)
Siblings			
Yes	51.6 (403)	49.6 (199)	47.3 (204)
Child’s living arrangements			
With both parents	94.7 (788)	94.5 (379)	94.9 (409)
Only or mostly with one parent	4.0 (33)	3.7 (15)	4.2 (18)
Joint physical custody*	1.3 (11)	1.7 (7)	0.9 (4)
Preschool			
Yes	34.3 (285)	32.4 (130)	36.0 (155)

^*^A living arrangement for children whose parents are separated or divorced. The child spends roughly equal amounts of time living with each parent

Analyses of the post-intervention attrition revealed that parents who were lost to follow-up were more likely to be born outside Sweden and had lower levels of education at a significantly higher rate (Supplementary Table S2, Additional File 2). There was higher attrition at post-assessment in the control group (68%) compared to the Little ACF group (32%). However, analyses of internal attrition revealed no significant differences in baseline sociodemographic characteristics between groups (Supplementary Table S3, Additional File 3).

### Procedure

All group leaders participated in three days of training including study-specific guidance on procedures and responsibilities within the RCT. The training sessions were held every other week to allow group leaders to run groups in between. Training comprised both theoretical content and practical exercises and was provided by experienced educators external to the research team. After each group session with families, group leaders completed checklists to document the session process and parental engagement. A research coordinator was available to group leaders throughout the study period via telephone, email, and a study-specific website, where questions were addressed and ongoing information about study progress was provided. Written informed consent was obtained from all the participating group leaders.

Parents were invited to participate in this study during their child’s routine 10-, 12 or 18-month visit to the CHS between August 2022 and May 2024. The CHS nurse informed all eligible parents about the study orally and provided them with an information leaflet. Potentially interested parents received an email with a link to a study-specific website. To participate, a consent form was signed and a baseline questionnaire on the website (CSAM) was completed. Participants provided informed consent and completed questionnaires using BankID, a secure Swedish electronic identification system widely used for online authentication and digital signatures. The baseline questionnaire contained background questions on the age and gender of the child and the parent, parental educational level, country of birth, and family composition as well as the study instruments. The primary outcome measures were focused on parenting, while child development served as a secondary outcome measure. Primary and secondary outcomes were repeated in the post-intervention questionnaire. No reminders were issued for the Little ACF group sessions or the control digital lectures, and no reimbursements were provided.

### Randomisation and masking

When 10–16 parents had completed the baseline questionnaires, the CHS nurse used the CSAM platform to randomise the child to one of the trial arms (i.e., parents of the same child were assigned to the same trial arm). In families with twins, one sibling was designated by the parents as the focus child for the study. To ensure concealment, the CSAM platform generated randomisation at a single point with no possibility for intervention by the CHS nurse. Information about allocation was sent automatically to the families by CSAM after randomisation.

Due to the nature of the intervention both participants and group leaders were aware of the two study arms and were therefore not blinded. Additionally, the researchers were not blinded during the intervention and data analyses.

### Interventions

#### Little ACF

The Little ACF programme consisted of four group sessions with different themes, with the core components being: (1) strengthening the parent–child relationships, (2) emotion regulation for parents and children, and (3) parenting strategies to be practiced at home between the group sessions. The sessions were intended to be scheduled on a weekly basis, although the interval between sessions sometimes varied slightly (e.g., 1 to 2 weeks) due to scheduling considerations. The group sessions lasted 60 min, followed by an additional 30 min of informal discussion and peer interaction. Given the young age of the children, parents were encouraged to bring them. The sessions incorporated short texts, illustrations, pre-formulated discussion questions and role-playing activities. The group leader manual followed a consistent structure across all four sessions, providing clear guidelines on the leaders’ responsibilities such as when to introduce specific questions. The first three sessions concluded with a practical example for parents to try at home, which was discussed during the following session. At the end of each session, parents received printed materials to support further discussion and sharing of the content with a potential coparent. For a more detailed summary of the content of the Little ACF programme, please see the study protocol [26].

#### Digital lectures

Parents who were assigned to the control condition received access to four pre-recorded digital lectures. Each lecture lasted between 10 and 19 min and provided information about parenting and child development. The lectures were informational and did not include actionable parenting strategies. For a more detailed account of the control group’s conditions, see the study protocol [26].

### Measurements

#### Primary outcome measures

An adapted Swedish version of *Tool to Measure Parenting Self-Efficacy* (TOPSE) was used to measure parenting self-efficacy [12, 31]. This 48-item scale assesses eight domains of parenting: emotion and affection, play and enjoyment, empathy and understanding, control, setting boundaries, pressures of parenting, self-acceptance, learning and knowledge. Parents indicate how much they agree with each statement on an 11-point Likert scale from 0 (completely disagree) to 10 (completely agree) with higher scores indicating higher self-efficacy. In the present study sample, internal reliability according to Cronbach’s alpha was excellent, with α = 0.93 both at baseline and post-intervention.

Two subscales from *The Swedish Parenthood Stress Questionnaire* (SPSQ) were used; incompetency and role limitation*,* as they represent the most psychometrically robust scales [32]. The 18 items include five response alternatives, ranging from 1 (not true at all) to 5 (very true) with higher scores indicating more stress. The Cronbach’s alpha in the study sample was α = 0.88 at both baseline and post intervention indicating very good reliability.

To assess the quality of parents’ coparenting relationship we included four questions inspired by *the Coparenting Relationship Scale* [33]*.* These items have been validated in a Swedish context [34]. The answers range from 1 (not true at all) to 5 (very true) with higher scores indicating higher coparenting quality. We found a Cronbach’s alpha of α = 0.82 at baseline and α = 0.83 post-intervention, indicating very good reliability.

The emotion regulation of the parent in relation to the child was measured using the *Parent Emotion Regulation Scale* (PERS) developed by Pereira et al. [35]. The 20-items scale assesses parents’ orientation to the child’s emotions, avoidance of the child’s emotions, emotional lack of control, and acceptance of the child’s and the parent’s emotions. The response scale ranges from 1 (never or almost never) to 5 (always or almost) with higher scores indicating better emotion regulation abilities. We found a Cronbach’s alpha of α = 0.53 at baseline and α = 0.55 post-intervention, indicating low reliability.

How parents handle young children’s negative emotions was assessed using the *Coping with Toddler’s Negative Emotions Scale* (CTNES) [9]. In its original version, 12 situations are described to which the parents should rate the likelihood of using 6–7 different strategies to solve each situation, representing different parenting strategies. The likelihood rating ranges from 1 (not likely at all) to 7 (very likely). For this study, 8 age-appropriate situations (i.e., item 3–10) were selected. According to Spinrad et al. we merged the supportive subscales expressive encouragement, emotion-focused reactions, and problem focused reactions to a scale named CTNES SUP, and the non-supportive subscales punitive reactions and minimisation reactions to a scale named CTNES NON-SUP [9]. Cronbach’s alpha was α = 0.87 at both baseline and at post-intervention for the CTNES supportive scale, and α = 0.84 at baseline and α = 0.85 at post-intervention for the non-supportive scale, indicating good reliability on both scales.

#### Secondary outcome measure

For measuring children’s social and emotional development we used *the Ages and Stages Questionnaire: Social Emotional – Second Edition* (ASQ:SE-2) [36]. In a recent psychometric validation study, our research group validated the ASQ:SE-2 for 18-month-old children and found the Swedish version to be inadequate in a community sample [37]. Acceptable reliability was found only among the approximately 25% of children exhibiting the highest levels of socioemotional difficulties. Hence, we only conducted analysis on children above the 75th percentile at baseline assessment. In the present study, we found Cronbach’s alphas of 0.71 at baseline and α = 0.69 post-intervention, indicating acceptable reliability.

#### Feasibility outcome

To assess attendance, group leaders recorded each participant’s presence at every session in the intervention group. The questionnaire also included items where parents indicated whether, and if so, which parenting strategies they had tried at home together with their child. For the control group, parents were asked whether they had viewed the digital lectures or not. For process evaluation, group leaders completed a checklist after each session. The checklist covered whether there was sufficient time for each module in the manual, whether they felt able to use their group facilitation skills, how the home exercises were received by the parents, and the level of parental engagement.

### Statistical analyses

Statistical analyses were conducted using the software SPSS and R (package lme4). The proportion of missing data on item level was low on all the instruments included (< 5%) and these were imputed using the average item score on the test. Family and child characteristics were summarised overall and by trial arms at baseline. To explore pre-post differences between groups, linear mixed models (LMM) were used, with assessments nested within parent, and parents nested within child. LMMs have been shown to provide unbiased estimates for intention-to-treat analyses without requiring imputation in most scenarios, making them an appropriate choice when follow-up data are missing [38]. Sensitivity analyses on unimputed data were conducted to ensure that our conclusions were not dependent on imputation method. In addition, we explored potential moderators of the effects using factors shown to boost the effects in the previous evaluation of the ACF for parents of children aged 3 to 12 years (i.e., parental gender, education level, country of birth, parenting stress, and child gender, age and presence of siblings). Moderation analyses were performed exploring three-way interactions (group × time × moderator) in the LMMs.

## Results

### Primary and secondary outcome measures

Primary outcomes concern parental measures of parenting self-efficacy, parenting stress, coparenting quality, parents’ emotion regulation, and strategies for regulating children’s negative emotions. Secondary outcome concern child measures of social-emotional development. Compared to the control group, parents in the Little ACF showed a significantly greater increase in the use of supportive strategies to regulate their children’s negative emotions, as reflected in a higher estimated difference from baseline to post intervention $(\beta$ = 3.0, CI = 0.9–5.0, Cohen’s *d* = 0.20, CI = 0.06–0.34). There was a tendency (non-significant) that parents in the Little ACF group showed higher self-efficacy $(\beta$= 3.8, CI = −0.3 – 8.0, Cohen’s *d* = 0.09, CI = −0.01 – 0.20). No statistically significant effects were observed on any of the other outcome measures (Table 2). Sensitivity analyses were conducted to ensure that the findings were not driven by the effects of imputed data but revealed no substantial differences in the observed effects (Supplementary Table S4, Additional File 4).

**Table 2:** Descriptive data on included outcome measures pre and post intervention and estimated difference (*N* = 832)

	*Intervention*				*Control*						
	*Pre*		*Post*		*Pre*		*Post*		*Estimated difference (Group x Time)*		
*Primary outcomes*	*M (SD)*	*n*	*M (SD)*	*n*	*M (SD)*	*n*	*M (SD)*	*n*	*Beta (95% CI)*	*Cohen’s d (95% CI)*	*P-value*
TOPSE	393.0 (42.4)	401	401.8 (39.1)	331	392.9 (44.1)	426	398.1 (42.6)	280	3.8 (−0.3, 8.0)	0.09 (−0.01,0.20)	0.072
SPSQ	49.8 (11.5)	401	49.2 (10.8)	334	50.6 (10.9)	430	50.0 (10.7)	289	0.3 (−0.8, 1.3)	0.02 (−0.08, 0.12)	0.629
COPARENTING	17.1 (3.0)	394	17.2 (2.7)	327	17.3 (2.8)	417	17.3 (2.8)	279	0.2 (−0.2, 0.5)	0.07 (−0.06, 0.19)	0.291
PERS	63.2 (6.4)	397	64.3 (6.2)	329	63.3 (6.2)	425	64.2 (5.7)	277	0.1 (−0.7,1.0)	0.02 (−0.13, 0.16)	0.809
CTNES SUP	145.8 (19.4)	397	152.8 (13.8)	327	146.4 (17.1)	424	149.7 (15.3)	273	3.0 (0.9, 5.0)	0.20 (0.06, 0.34) **	0.005**
CTNES NON-SUP	32.0 (12.4)	397	30.1 (11.6)	326	33.0 (11.8)	424	31.8 (12.5)	273	−0.7 (−2.0, 0.6)	−0.06 (−0.17, 0.05)	0.265
*Secondary outcome*											
ASQ:SE-2^a^	25.8 (19.0)	401	18.9 (15.5)	331	24.5 (17.6)	430	17.6 (16.1)	283	−0.9 (−7.0, 5.3)	−0.05 (−0.37, 0.27)	0.781

Calculated on imputed values. *TOPSE* Tool to Measure Parenting Self-Efficacy, *SPSQ* The Swedish Parenthood Stress Questionnaire, *Coparenting* Coparenting Relationship Scale, *PERS* Parent Emotion Regulation Scale, *CTNES SUP* Coping with Toddlers’ Negative Emotions Scale Supportive, *CTNES NON-SUP* Coping with Toddlers’ Negative Emotions Scale Non-Supportive, *ASQ:SE-2* Ages and Stages Questionnaire: Social Emotional – Second Edition

^a^Analysis only conducted on the 25% of children with highest levels of socioemotional difficulties

^*^ *p* < 0.05

^**^ *p* < 0.01

^***^ *p* < 0.001

Overall, no significant moderation effects were found, but there was a trend towards a stronger impact on fathers' use of positive strategies to manage their children's negative emotions compared with mothers ($\beta$ = −4.06, CI = −8.47 – 0.34) (Supplementary Table S5, Additional File 5).

### Feasibility outcome

The third research question concerned the feasibility of the program, specifically whether Little ACF could engage and retain parents in group sessions. Approximately 70% of parents in the Little ACF attended three or all four sessions, while 7.5% did not participate at all. Participation rates across the four occasions ranged from 66.8% to 76.1% (Supplementary Table S6, Additional File 6). Adherence to parenting strategies assigned for home practice between group sessions was high, with approximately 90% of parents reporting that they had practiced the strategies at home. In the control group, slightly more than 50% viewed the digital lectures. Of those who completed the T2 assessment, a larger proportion (76%) reported having viewed the lectures. Process evaluation indicated high adherence to the method among group leaders, with sufficient time to cover all sections of the manual and effective use of leadership skills. According to group leaders, parents responded positively to the home practice instructions, and parental engagement was strong (data not shown).

## Discussion

This two-arm parallel RCT evaluated immediate post-intervention effects of the Little ACF programme on parenting and child outcomes, in comparison with four digital lectures on child development. Parents in the intervention group reported significantly greater use of supportive strategies for regulating their children’s negative emotions, alongside a trend toward enhanced parenting self-efficacy. No significant differences were observed across other outcomes. The intervention appeared feasible, with high levels of parental engagement and retention across sessions, as well as strong adherence to home practice tasks. This may add to existing knowledge about feasibility factors important for long-term outcomes in universal interventions [13, 24]. The observed increase in supportive parenting strategies in our study connects directly to previous research emphasizing that a central aspect of parenting during the first three years is to foster children’s emotion regulation through parental practices [6–8]. In line with these findings, our results suggest that parents’ strategies for responding to children’s negative emotions are critical, as they shape children’s opportunities to develop adaptive regulation [39]. Our findings also partially align with previous research on the ACF program for parents of children aged 3–12 years [12, 25]. Similar to these earlier studies, we observed increased use of emotion regulation skills. In contrast, improvements in parenting self‑efficacy were less pronounced, showing only a tendency towards being strengthened. One possible explanation may be that parents of 1–2‑year‑olds have relatively higher levels of parenting confidence due to fewer behavioural and everyday challenges than parents of 3–12-year-olds. In Sweden, this early developmental period is often experienced as comparatively stable, with many families still on parental leave and caring for only one child, which may contribute to already high initial levels of perceived competence and therefore limit the scope for measurable change.

Turning to the null findings for parenting stress, coparenting quality, and parents’ own emotion regulation, several factors may help explain these results. As suggested in previous meta-analytic reviews by Jeong et al. [14] and Wyatt Kaminski et al. [19], Little ACF included components such as supportive parenting strategies and opportunities for parents to practise new skills. Parents were encouraged to practise the three included emotion regulation strategies with their children between the sessions, while parenting stress and coparenting quality were addressed through short lectures and group discussions. It is possible that strategies for regulating children’s emotions were more concrete and easier to implement than those aimed at parents’ own emotion regulation. Enhancing parent emotion regulation may require more time, self-reflection and new experiences. Further, given that parenting stress is influenced by multiple factors, including social support, domestic workload, and fathers’ engagement, change may take longer to manifest [33, 40]. This aligns with findings from the CANparent Trial [11], which reported no reduction in parenting stress despite improvements in parenting self-efficacy and mental well-being. The Australian universal programme TOTS, which targets parents of toddlers and has aims aligning with those of the Little ACF, found improved parent emotion socialization (i.e. the process through which children learn about emotions) and child functioning at a 12-month follow-up [22]. These results suggest that emotional and behavioural shifts in parents and children may require extended time to emerge. Such changes are likely to begin with gradual adjustments in parents’ own regulation and interaction patterns, which over time may influence children’s development.

The observed effect size (Cohen’s *d* = 0.20) for supportive emotion regulation strategies can be considered small according to conventional benchmarks. However, in the context of a universal intervention targeting parents of young children, this level of change may represent a meaningful impact. Many parents in such programs already employ supportive strategies, which may limit the scope for large changes [10, 41]. Although the control condition excluded parental strategies, the digital lectures may still have offered informative support, qualifying as a moderately active control condition that was perhaps more effective than anticipated. Furthermore, while the effect was modest at the group level, increases in supportive emotion regulation strategies may still be meaningful for individual families. It is also widely acknowledged that conventional benchmarks for effect sizes should not be applied rigidly across different populations, interventions, or prevention levels. In the context of a universal intervention targeting parents of young children, before difficulties have fully emerged, even small effects can be of preventive value [42]. Additionally, universal interventions carry potential to reduce stigma and exclusion, particularly among parents facing mental health, socioeconomic, or identity-related vulnerabilities, as emphasized in international guidelines [27, 28]. However, this was not the case in the present study, which primarily included well-educated, Swedish-born parents. Such selective participation is common in RCTs within the field of parenting support interventions, where samples often reflect more advantaged groups [13]. Nevertheless, subsequent dissemination of the ACF programme for parents of children aged 3–12 years in Sweden has reached a broader and more representative segment of the population, indicating that universal approaches can be implemented in ways that extend beyond the initial trial sample [43].

The low drop-out rate suggests that the Little ACF programme successfully engaged and retained parents. Adherence to the parenting strategies practiced at home between group sessions was also high, with approximately 90% of parents practicing the tasks together with their children. This indicates strong engagement with the intervention, which may have contributed to the high retention of parents across the group sessions.

Beyond the specific outcomes reported in this study, additional insights emerged during supervision, training sessions, and from parents’ and group leaders’ open responses. Although these were not part of the systematic analyses, they suggest that group leaders, well prepared through the training, fostered a safe and rewarding atmosphere; that parents perceived the content as relevant and the strategies as useful; and that collaboration between child health services and municipalities/civil society functioned well. Taken together, these observations indicate that the intervention can be successfully implemented within the CHS context and may further inform feasibility considerations for universal parenting interventions.

### Limitations

Beyond the limitation that our sample was not representative of the general population [44], attrition analyses further showed that parents who dropped out were more often born outside Sweden and had lower education. Future development of the Little ACF programme should therefore focus on reaching families with varying educational backgrounds and countries of origin. Another constraint is the use of an instrument to assess children’s socioemotional development (ASQ:SE-2) that was shown not to capture variation within the general population [37]. In a previous validation study, the ASQ:SE-2 for 18-month-old children was found to be reliable only among children with difficulties (i.e., above the 75th percentile). Consequently, analyses of socioemotional development were restricted to children scoring above the 75th percentile (i.e., those with greater difficulties). This limitation means that we cannot draw conclusions about the intervention’s effect on socioemotional development for the overall study sample. In addition, the measure of parent emotion regulation (PERS) demonstrated low internal reliability below the commonly accepted threshold. Furthermore, the parental outcome measures on parenting stress and coparenting quality may not have fully reflected the specific content and aims of the intervention. More generally, there are few measures available for 1–2-year-old children within these domains that are well adapted and validated for Swedish conditions, which may have constrained our ability to observe effects.

### Strengths

The large sample size is a methodological strength of the present study. Also the use of instruments to assess positive outcomes, which is particularly relevant in early health promotion when major child difficulties may not yet have emerged, may be regarded as a strength. We assessed parenting stress and negative strategies given their known adverse impact on child development. Although no statistically significant effects were observed on these aspects in this pre-post study, Little ACF may still help identify families in need of additional supportive interventions at an early stage.

Another strength is that nearly 30% of participants were fathers, which is a relatively high proportion in a field largely focused on mothers [14–16]. However, this also highlights the continued underrepresentation of fathers, underscoring the need for continued efforts to ensure that parental support is designed and delivered in ways that reach all caregivers, regardless of gender.

### Future research

Several directions for future research emerge from these findings. A key priority is to explore how universal parenting programmes like Little ACF can be made accessible and effective for families with diverse educational and cultural backgrounds. This includes examining strategies for cultural adaptation, translation of materials, and structural adjustments that facilitate participation among underrepresented groups. Evidence from the ACF programme for parents with children aged 3–12 years, a universal parenting programme with aims similar to those of Little ACF, indicates that such reach can be expanded over time, with subsequent implementation achieving a more representative participation beyond the initial RCT sample [43]. Moreover, given that CHS in Sweden reach nearly all families with young children, this platform provides promising opportunities to extend the reach of universal parenting programmes even further. Additional research is also needed to investigate the mechanisms underlying changes in parenting practices and child outcomes, as well as the long-term impact of universal approaches delivered through collaborative partnerships between CHS and municipalities or civil society organisations. The forthcoming follow-ups of Little ACF will provide valuable insights into whether the measured aspects of parenting continue to evolve over time or whether the immediate outcomes may attenuate. Continued monitoring may also clarify whether children with the greatest social and emotional developmental difficulties can benefit from Little ACF over time.

## Conclusions

The universal Little ACF programme demonstrates potential to promote supportive strategies for regulating children’s negative emotions alongside a tendency toward enhanced parenting self-efficacy among parents of 1–2-year-olds immediately post intervention. No statistically significant effects were observed for parenting stress, coparenting quality, parents’ own emotion regulation, or child development. The results demonstrate that the program had a strong capacity to engage and retain parents in group settings. These findings contribute to the limited evidence base on early universal interventions and suggest that even brief, child-inclusive interventions may affect parenting behaviours relevant to children’s positive development. Long-term effects remain to be determined through forthcoming follow-up assessments.

## Supplementary Information

Additional file 1: Supplementary Table S1. Reasons for exclusion from study, shown in frequencies and percentages (N=322).

Additional file 2: Supplementary Table S2. Analyses of internal attrition based on sociodemographic characteristics between study sample (N=623) and those excluded (N=209), presented as percentages, means, standard deviations and test difference.

Additional file 3: Supplementary Table S3. Analyses of internal attrition based on sociodemographic characteristics between intervention and control groups, presented as percentages, means, standard deviations and test difference (N=209).

Additional file 4: Supplementary Table S4. Estimated difference on included instruments with non-imputed values (N=832).

Additional file 5: Supplementary Table S5. Moderating effects of parent’s and child characteristics on TOPSE and CTNES (N=832).

Additional file 6: Supplementary Table S6. Participation rates in the Little ACF group, shown in percentages and number of participants (n=401).

## Data Availability

Due to the ethical approval which states that the information from participating parents and CHS nurses should respect anonymity, all data is only accessed by the research team and stored on secure platforms at Karolinska Institutet. Access to pseudonymised trial data can be granted by the corresponding author upon reasonable request, as long as the request aligns with the data sharing regulations of Karolinska Institutet and adheres to Swedish legal requirements.

## Abbreviations

ACF: All Children in Focus
ASQ:SE-2: Ages & Stages Questionnaires: Social-Emotional, Second Edition
CHS: Child Health Services
CSAM: The e-health platform used in the study for randomisation and data collection
CTNES SUP: Coping with Toddlers’ Negative Emotions Scale supportive
CTNES NON-SUP: Coping with Toddlers’ Negative Emotions Scale non-supportive
LMM: Linear Mixed Model
PERS: Parent Emotion Regulation Scale
RCT: Randomised Controlled Trial
SPSQ: Swedish Parenthood Stress Questionnaire
TOPSE: A Tool to measure Parenting Self Efficacy
