# Supplementary material for: Little All Children in Focus (Little ACF) – evaluation of a parenting support programme for parents of children aged 1–2 years in Sweden: a randomised controlled trial
Source: BMC Public Health. 2026 May 19;26:1563. doi: 10.1186/s12889-026-27733-2 (PMC13188431; doi:10.1186/s12889-026-27733-2)
Supplement: Supplementary file 1 [file 12889_2026_27733_MOESM1_ESM.odt]

Supplementary Table S1. Reasons for exclusion from study, shown in frequencies and percentages (N=322)

| Reason | Frequency (n) | Percent (%) |
| --- | --- | --- |
| Did not consent despite reminders | 96 | 29.8 |
| Start work/studies or preschool | 33 | 10.2 |
| Declined | 86 | 26.7 |
| Technical issues | 10 | 3.1 |
| Other | 97 | 30.2 |
| Total | 322 | 100.0 |
