# Supplementary material for: Little All Children in Focus (Little ACF) – evaluation of a parenting support programme for parents of children aged 1–2 years in Sweden: a randomised controlled trial
Source: BMC Public Health. 2026 May 19;26:1563. doi: 10.1186/s12889-026-27733-2 (PMC13188431; doi:10.1186/s12889-026-27733-2)
Supplement: Supplementary file 2 [file 12889_2026_27733_MOESM2_ESM.odt]

Supplementary Table S2. Analyses of post-intervention attrition based on sociodemographic characteristics between study sample (N=623) and those excluded (N=209), presented as percentages, means, standard deviations and test difference 

|  |  | Study sample n (%) | Attrition n (%) | Test difference Chi2/t-test (p) |  |
| --- | --- | --- | --- | --- | --- |
|  | Respondent |  |  |  |  |
|  | Mother | 429 (68.9) | 149 (71.3) |  |  |
|  | Father | 194 (31.1) | 60 (28.7) | 0.51 |  |
|  | Parental age in years (mean, SD) |  |  |  |  |
|  | Mothers | 34.8 (4.7) | 34.7 (5.2) |  |  |
|  | Fathers | 37.5 (5.0) | 35.9 (4.9) | 0.14 |  |
|  | Parental country of birth |  |  |  |  |
|  | Sweden | 523 (83.9) | 131 (74.6) |  |  |
|  | Other country | 100 (16.1) | 78 (25.4) | 0.003* |  |
|  | Parental educational level |  |  |  |  |
|  | High school or lower | 172 (27.6) | 78 (37.3) |  |  |
|  | University/college | 451 (72.4) | 131 (62.7) | 0.008* |  |
|  | Previous parental group |  |  |  |  |
|  | Yes | 222 (35.6) | 77 (36.8) |  |  |
|  | No | 401 (64.4) | 132 (63.2) | 0.75 |  |
|  | Child’s sex |  |  |  |  |
|  | Girl | 323 (51.8) | 110 (52.6) |  |  |
|  | Boy | 300 (48.2) | 99 (47.4) | 0.84 |  |
|  | Child’s age in months (mean, SD) | 15.8 (5.9) | 15.7 (5.2) | 0.82 |  |
|  | Siblings |  |  |  |  |
|  | Yes | 293 (47.0) | 110 (52.6) |  |  |
|  | No | 330 (53.0) | 99 (47.4) | 0.16 |  |
|  | Child’s living arrangements |  |  |  |  |
|  | With both parents | 591 (94.9) | 197 (94.3) |  |  |
|  | Other living arrangementsa | 32 (5.1) | 12 (5.7) | 0.74 |  |
|  | Preschool |  |  |  |  |
|  | Yes | 203 (32.6) | 82 (39.2) |  |  |
|  | No | 420 (67.4) | 127 (60.8) | 0.08 |  |
| aDue to small sample size the categories ‘Only or mostly with one parent’ and ‘Joint physical custody’ are merged. *Significance on 0.01 level. |
