# Supplementary material for: Little All Children in Focus (Little ACF) – evaluation of a parenting support programme for parents of children aged 1–2 years in Sweden: a randomised controlled trial
Source: BMC Public Health. 2026 May 19;26:1563. doi: 10.1186/s12889-026-27733-2 (PMC13188431; doi:10.1186/s12889-026-27733-2)
Supplement: Supplementary file 3 [file 12889_2026_27733_MOESM3_ESM.odt]

Supplementary Table S3. Analyses of internal attrition based on sociodemographic characteristics between intervention and control groups, presented as percentages, means, standard deviations and test difference (N=209) 

|  | Intervention n (%) | Control n (%) | Test difference Chi2/t-test (p) |
| --- | --- | --- | --- |
| Overall Respondent | 67 (32.0) | 142 (68.0) | 0.000* |
| Mother | 48 (71.6) | 101 (71.1) |  |
| Father | 19 (28.4) | 41 (28.9) | 1.00 |
| Parental age in years (mean, SD) |  |  |  |
| Mothers | 35.2 (4.8) | 34.5 (5.3) |  |
| Fathers | 35.4 (4.0) | 36.2 (5.3) | 0.12 |
| Parental country of birth |  |  |  |
| Sweden | 46 (68.7) | 110 (77.4) |  |
| Other country | 21 (31.3) | 32 (22.6) | 0.23 |
| Parental educational level |  |  |  |
| High school or lower | 26 (38.8) | 52 (36.6) |  |
| University/college | 41 (61.2) | 90 (63.4) | 0.88 |
| Previous parental group |  |  |  |
| Yes | 23 (34.3) | 54 (38.0) |  |
| No | 44 (65.7) | 88 (62.0) | 0.61 |
| Child’s sex |  |  |  |
| Girl | 42 (62.7) | 68 (59.4) |  |
| Boy | 25 (37.3) | 74 (40.6) | 0.62 |
| Child’s age in months (mean, SD) | 16.0 (5.4) | 15.5 (5.1) | 0.95 |
| Siblings |  |  |  |
| Yes | 35 (52.2) | 75 (52.8) |  |
| No | 32 (47.8) | 67 (47.2) | 0.94 |
| Child’s living arrangements |  |  |  |
| With both parents | 61 (91.0) | 136 (95.8) |  |
| Other living arrangements | 6 (9.0) | 6 (4.2) | 0.17 |
| Preschool |  |  |  |
| Yes | 26 (38.8) | 56 (39.4) |  |
| No | 41 (61.2) | 86 (60.6) | 0.93 |
| Note: *Significant on a 0.001 level. |  |  |  |
