# Supplementary material for: Little All Children in Focus (Little ACF) – evaluation of a parenting support programme for parents of children aged 1–2 years in Sweden: a randomised controlled trial
Source: BMC Public Health. 2026 May 19;26:1563. doi: 10.1186/s12889-026-27733-2 (PMC13188431; doi:10.1186/s12889-026-27733-2)
Supplement: Supplementary file 4 [file 12889_2026_27733_MOESM4_ESM.odt]

Supplementary Table S4. Estimated difference on included instruments with non-imputed values (N=832)

|  |  |
| --- | --- |
|  | Estimated difference (Group x Time) |
| Instrument | Beta | 95% CI | Cohen’s d |
| TOPSE | 3.76 | -0.54, 8.07 | 0.09 |
| SPSQ | 0.26 | -0.32, 0.85 | 0.02 |
| COPARENTING | 0.22 | -0.16, 0.60 | 0.07 |
| PERS | 0.43 | -0.40, 1.26 | 0.07 |
| CTNES SUP | 2.95 | 0.91, 4.99 | 0.20** |
| CTNES NON-SUP | -0.74 | -2.03, 0.56 | -0.06 |
| ASQ:SE-2a | 1.28 | -3.87, 6.44 | 0.08 |

Notes: TOPSE = Tool to Measure Parenting Self-Efficacy; SPSQ = The Swedish Parenthood Stress Questionnaire; Coparenting = Coparenting Relationship Scale; PERS = Parent Emotion Regulation Scale; CTNES SUP = Coping with Toddlers’ Negative Emotion Scale Supportive; CTNES NON-SUP = Coping with Toddlers’ Negative Emotion Scale Non-Supportive; ASQ:SE-2 = Ages and Stages Questionnaire: Social Emotional – Second Edition. 

aAnalysis only conducted on the 25% of children with highest levels of socioemotional difficulties.

* p< 0.05; ** p< 0.01; *** p< 0.001
