# Supplementary material for: Little All Children in Focus (Little ACF) – evaluation of a parenting support programme for parents of children aged 1–2 years in Sweden: a randomised controlled trial
Source: BMC Public Health. 2026 May 19;26:1563. doi: 10.1186/s12889-026-27733-2 (PMC13188431; doi:10.1186/s12889-026-27733-2)
Supplement: Supplementary file 5 [file 12889_2026_27733_MOESM5_ESM.odt]

Supplementary Table S5. Moderating effects of parent’s and child characteristics on TOPSE and CTNES (N=832)

| Moderator | Outcome | Coefficient | 95% CI |
| --- | --- | --- | --- |
| Parents |  |  |  |
| Gender |  |  |  |
|  | TOPSE | 0.29 | -8.74, 9.33 |
|  | CTNES-SUP | -4.06 | -8.47, 0.34 |
| Educational level |  |  |  |
|  | TOPSE | -2.14 | -11.50, 7.22 |
|  | CTNES-SUP | 0.17 | -4.42, 4.75 |
| Country of birth |  |  |  |
|  | TOPSE | 0.74 | -10.70, 12.16 |
|  | CTNES-SUP | 3.96 | -1.68, 9.61 |
| Parental stress (SPSQ) |  |  |  |
|  | TOPSE | 0.18 | -8.12, 8.48 |
|  | CTNES-SUP | 0.39 | -3.71, 4.48 |
| Children |  |  |  |
| Gender |  |  |  |
|  | TOPSE | 5.66 | -2.65, 13.99 |
|  | CTNES-SUP | 0.17 | -3.91, 4.25 |
| Age in months |  |  |  |
|  | TOPSE | -0.01 | -0.72, 0.71 |
|  | CTNES-SUP | -0.20 | -0.55, 0.15 |
| Presence of siblings |  |  |  |
|  | TOPSE | 0.91 | -7.45, 9.27 |
|  | CTNES-SUP | 1.62 | -2.47, 5.70 |
|  |  |  |  |
