# Supplementary material for: Little All Children in Focus (Little ACF) – evaluation of a parenting support programme for parents of children aged 1–2 years in Sweden: a randomised controlled trial
Source: BMC Public Health. 2026 May 19;26:1563. doi: 10.1186/s12889-026-27733-2 (PMC13188431; doi:10.1186/s12889-026-27733-2)
Supplement: Supplementary file 6 [file 12889_2026_27733_MOESM6_ESM.odt]

Supplementary Table S6. Participation rates in the Little ACF group, shown in percentages and number of participants (n=401)

|  | Little ACF group |
| --- | --- |
|  | % (n) |
| Participation in specific meetings |  |
| 1 | 73.1 (293) |
| 2 | 76.1 (305) |
| 3 | 69.6 (279) |
| 4 | 66.8 (268) |
| Participation in number of meetings |  |
| 0 | 7.5 (30) |
| 1 | 7.0 (28) |
| 2 | 16.0 (64) |
| 3 | 31.7 (127) |
| 4 | 37.9 (152) |
